# The brain after COVID-19: Compensatory neurogenesis or persistent neuroinflammation?

**DOI:** 10.1016/j.eclinm.2020.100684

**Published:** 2020-12-24

**Authors:** Elkhonon Goldberg, Kenneth Podell, Daniel K. Sodickson, Els Fieremans

**Affiliations:** a Department of Neurology, NYU Grossman School of Medicine, 315 West 57th Street, Suite 401, New York, NY 10019, USA; b Stanley H. Appel Department of Neurology, Houston Methodist and Weill Cornell Medical College, 6560 Fannin Street, Suite 1840, Houston, TX 77030 USA; c Department of Radiology, NYU Grossman School of Medicine, 660 First Avenue, Fourth Floor, Room 407, New York, NY 10016, USA; d Department of Radiology, NYU Grossman School of Medicine, 660 First Ave. 2nd floor, 205, New York City, NY 10016, USA

Yiping Lu et al. [1] report increased grey matter volumes and changes in MRI-based measures of water diffusion in white matter in the brains of recovered COVID-19 patients three months after acute illness, compared to healthy controls. They propose that neurogenesis and hypertrophy caused volumetric enlargement, and pathway remyelination restricted diffusion in the patients. If valid, these explanations suggest vigorous and counter-intuitive compensatory brain mechanisms during recovery from COVID-19. However, both the methodology and the findings of the study allow alternative explanations.

The samples were matched for age and sex, but not for educational or premorbid neurocognitive variables known to correlate with microstructural and volumetric variables [2]. This allows the possibility that the patient sample included larger and more interconnected premorbid brains than the control sample.

Regional volume increase and decreased diffusivity may reflect persistent neuroinflammation rather than neurogenesis. Viral neuro-infections cause inflammatory states, activating several CNS cell types and causing edema [3], leading to transient increases in regional brain volume. Instead of remyelination, increased fractional anisotropy (FA) and decreased diffusivity may reflect axonal degeneration[4] or microgliosis and inflammation [5]. Cytotoxic edema may restrict diffusion; vasogenic edema may lead to reversible increases in FA. Overall, DTI is sensitive but not specific to changes in microstructure.

Different explanations of the findings lead to very different, even opposite, conclusions. The explanation proposed by Lu et al. implies vigorous neuro-compensatory processes in COVID-19. The alternative explanation, linking the volumetric and diffusivity findings to persistent neuroinflammation, highlights the persistent nature of neurological sequelae of COVID-19 long after resolution of acute illness.

## Declaration of Competing Interest

Dr. Goldberg reports personal fees from Eyeclick, personal fees from NESTRE, other from Oxford University Press, other from Penguin Random House Press, other from Elsevier, outside the submitted work.

Dr. Podell has nothing to disclose.

Dr. Sodickson reports personal fees and other from Q Bio, personal fees from Bruker, non-financial support and other from Siemens, non-financial support and other from Facebook, outside the submitted work; .

Dr. Fieremans reports personal fees from General Electric Company, other from MicSi, Inc., outside the submitted work.
